# CARRT—Motion Capture Data for Robotic Human Upper Body Model

**DOI:** 10.3390/s23208354

**Published:** 2023-10-10

**Authors:** Urvish Trivedi, Redwan Alqasemi, Rajiv Dubey

**Affiliations:** Department of Mechanical Engineering, University of South Florida, Tampa, FL 33620, USA; alqasemi@usf.edu (R.A.); dubey@usf.edu (R.D.)

**Keywords:** motion capture system, activities of daily living, OpenSim, Robotic Human Upper Body Model

## Abstract

In recent years, researchers have focused on analyzing humans’ daily living activities to study various performance metrics that humans subconsciously optimize while performing a particular task. In order to recreate these motions in robotic structures based on the human model, researchers developed a framework for robot motion planning which is able to use various optimization methods to replicate similar motions demonstrated by humans. As part of this process, it will be necessary to record the motions data of the human body and the objects involved in order to provide all the essential information for motion planning. This paper aims to provide a dataset of human motion performing activities of daily living that consists of detailed and accurate human whole-body motion data collected using a Vicon motion capture system. The data have been utilized to generate a subject-specific full-body model within OpenSim. Additionally, it facilitated the computation of joint angles within the OpenSim framework, which can subsequently be applied to the subject-specific robotic model developed MATLAB framework. The dataset comprises nine daily living activities and eight Range of Motion activities performed by ten healthy participants and with two repetitions of each variation of one action, resulting in 340 demonstrations of all the actions. A whole-body human motion database is made available to the public at the Center for Assistive, Rehabilitation, and Robotics Technologies (CARRT)-Motion Capture Data for Robotic Human Upper Body Model, which consists of raw motion data in .c3d format, motion data in .trc format for the OpenSim model, as well as post-processed motion data for the MATLAB-based model.

## 1. Introduction

In recent years, the field of robotics has undergone notable advancements and emerged as a forefront technology driven by artificial intelligence and machine learning. In order to meet the diverse needs of these applications, robotic systems have evolved in terms of structural design, dexterity, manipulability, adaptability, and intelligence. The robotics community has shown substantial interest in utilizing robots in personal and social environments [1,2,3]. Studies have emphasized the significance of predictability and motion velocity in robotic manipulators, as these factors significantly impact the performance of human collaborators [4,5,6]. One aspect of this development is investing in robotic manipulators with human-like motion characteristics. By achieving this, robots become more than machines; they become intuitive collaborators who can anticipate and synchronize their actions with human co-workers. This advancement holds immense promise in collaborative settings, mitigating anxiety and enhancing situational awareness among human collaborators. As a result, collaborative tasks within shared workspaces become more efficient, safer, and more conducive to productive interactions [5,7]. Therefore, it becomes important for researchers to formulate robust motion planning algorithms capable of accurately mimicking human movements to foster this sense of security. To gain a deeper understanding of human behavior during various daily living activities and to evaluate the effectiveness of algorithms, researchers need to have access to comprehensive datasets that include human motion data. Creating datasets encompassing comprehensive information for learning various performance metrics based on human demonstrations requires substantial effort. The data collection process involves capturing systematic movements of human motion. Comprehensive robotic models are constructed using data derived from anthropomorphic models for Opensim Version 3.3 and MATLAB R2019b software, along with the development of tools for processing and interpreting these datasets for the MATLAB environment. The availability of such datasets, including anthropomorphic models and post-processed datasets for various environments, represents a unique contribution that can significantly advance the field of Human Motor Control through biomimetic approaches.

In this paper, we present a dataset encompassing the following components:

1. Motion Capture Dataset: The dataset includes recordings captured using a VICON motion capture system, comprising a total of nine activities of daily living which includes eight unimanual activities and one bimanual activity of daily living and eight Range of Motion activities. These activities were performed by a group of ten individuals.
2. OpenSim Motion Files: For each of the tasks in the dataset, motion files compatible with OpenSim are provided. These files enable the study and analysis of the captured motions using OpenSim software.
3. An Upper Body Model for MATLAB: An approximate upper body model is included, specifically designed for MATLAB utilizing Peter Corke Robotic Toolbox. This model facilitates the investigation of various performance metrics utilizing MATLAB.
4. Post-processed Dataset: The dataset has undergone further processing to enable the exploration of a wide range of performance metrics within the MATLAB environment. This processed dataset allows researchers to conduct in-depth analyses and investigations.

The motion capture data have been carefully processed and presented in .xls format, allowing them to be used on different software platforms. However, in this dataset, the authors have provided all the required components for the MATLAB framework, making it easier to use the data within this specific environment. This dataset can be used to aid future research and advancements in the field of Human Motor Control using biomimetic approaches. To facilitate accessibility and utilization, the dataset is made publicly available through our CARRT—Motion Capture Data for Robotic Human Upper Body Model [8].

## 2. Related Work 

### Human Motion Datasets

In the field of biomimetic motion, many methods use human motion recordings as a basis and benchmark for assessment. This dependence on motion capture remains consistent regardless of the scope, field, or application being considered. However, the limited accessibility to large, adaptable datasets of high quality, negatively impacts the overall extent of research in this area [9]. An approximate human kinematic model is crucial to conduct a comprehensive kinematic analysis. This model encompasses the joint angles and movements exhibited by the human body during different activities. Despite multiple motion capture databases, it is crucial to highlight that most of these researchers have tailored their datasets to suit their specific research requirements. As a result, their limited coverage of daily living activities makes them less helpful in conducting task-specific kinematic analysis.

The KIT Whole-Body Human Motion Database [10] is a comprehensive repository of large-scale whole-body human motion data. It provides a range of methods and tools that facilitate a unified representation of captured human motion and enable efficient searching within the database. Moreover, it allows for the transfer of subject-specific motions to robots with varying physical characteristics. In addition to detailing the reference model, the authors of the database outline systematic procedures and techniques for recording, labeling, and organizing human motion capture data. They also address the recording of object motions and the establishment of subject–object relations. The AndyData-lab-onePerson Dataset [11] comprises a collection of datasets that include motion and force measurements captured during various manual tasks. The dataset also includes detailed annotations of the actions and postures exhibited by the participants during these tasks. In this dataset, a total of 13 participants were involved, each engaging in a series of activities that simulate industrial tasks commonly encountered in real-world settings. These include tasks such as setting screws at different heights and manipulating loads of varying weights.

The CMU Graphics Lab Motion Capture Database [12] is a prominent repository of human motion data. It comprises 6 major task categories, each further divided into 23 subcategories, resulting in a total of 2605 trials. This database offers a diverse range of captured motions, encompassing various activities and scenarios such as walking, washing clothes, playing games, etc. 

The SFU Motion Capture Database [13] has significantly contributed to the field by offering a comprehensive dataset encompassing a wide range of human motions. The dataset includes recordings of eight subjects performing tasks across five major categories, providing researchers with valuable insights into human movement patterns and behavior. One notable advantage of the SFU Motion Capture Database is its provision of motion data in multiple file formats. This versatility lets users import motion files into various platforms, including Maya, MotionBuilder, and OpenSim. 

The Archive of Motion Capture As Surface Shapes (AMASS) [14] is a comprehensive and diverse database of human motion. It serves as a unifying platform for integrating data from 15 distinct optical marker-based motion capture (mocap) datasets, employing a shared framework and parameterization approach. The authors of the study utilized a software tool called MoSh++ to transform the mocap data into realistic three-dimensional (3D) human meshes, represented by a rigged body model. 

The GRAB (GRasping Actions with Bodies) dataset [15] offers an extensive repository of whole-body grasping actions, encompassing complete three-dimensional (3D) shape and pose sequences of 10 subjects interacting with 51 everyday objects exhibiting diverse shapes and sizes. However, it is important to emphasize that the dataset’s primary research focuses on object grasping rather than capturing the intricacies of actual object interactions. Furthermore, the dataset represents the body using the SMPL-X model, which needs more detailed human body parameters for conducting thorough kinematic analyses.

In contrast to previous research, our study introduces a task-specific motion capture dataset that captures whole-body motions during daily living tasks. This dataset is adaptable across different platforms and seamlessly integrates subject-specific kinematic models into the MATLAB workspace. This dataset and these models play a crucial role in conducting diverse analyses to understand the various performance criteria that humans intuitively optimize during the execution of specific activities of daily living tasks. This dataset serves as a valuable complement to the previously established KIT Whole-Body Human Motion Database [10] and the GRAB (GRasping Actions with Bodies) dataset [15].

## 3. The Dataset

The primary objective of this section is to provide a comprehensive description of the marker placement and camera setup employed in the study, as well as detailed participant demographics and the experimental setup. Moreover, it encompasses a comprehensive description of the objects utilized during the performance of various activities of daily living. The CARRT-Motion Capture Data for Robotic Human Upper Body Model Database offers an extensive assortment of anthropomorphic data, .c3d motion files, and a subject-specific Robotic-Toolbox-based MATLAB model.

### 3.1. Marker Placement and Camera Setup

**Figure 1:**
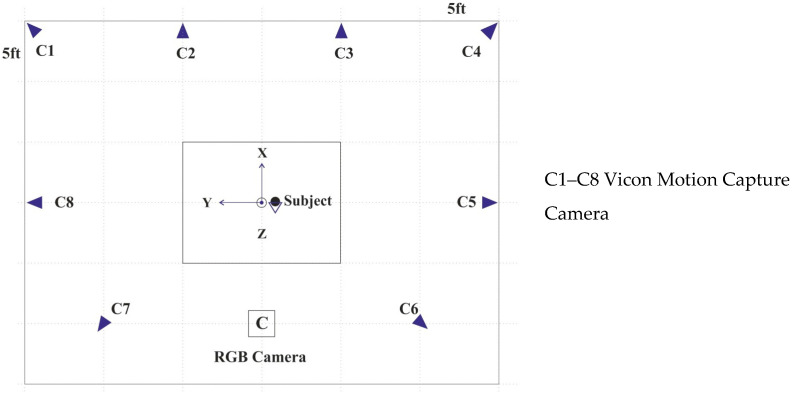
Camera positions relative to subject and motion capture system origin.

**Figure 2:**
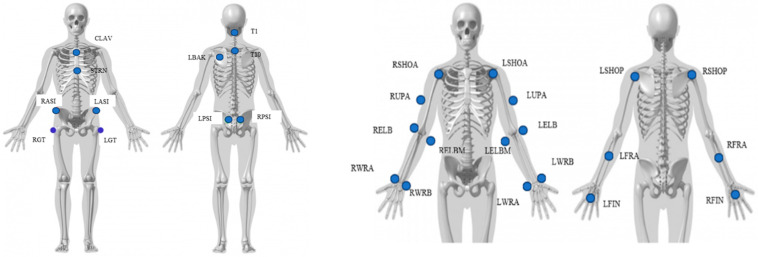
Upper body marker placement.

**Figure 3:**
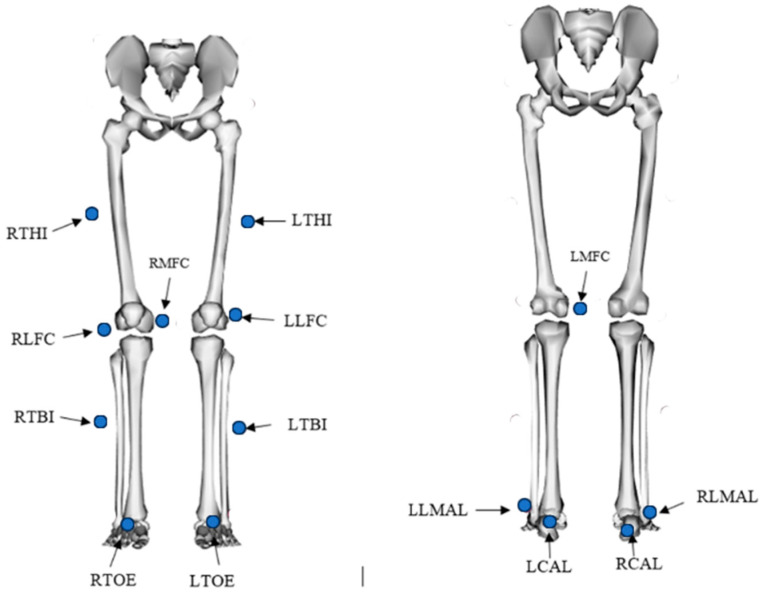
Lower body marker placement.

In this dataset, the entire kinematics of the participant’s whole body was captured using a Vicon motion capture system based on reflective markers [16]. Eight cameras were strategically positioned around the workspace, as shown in Figure 1. For marker placement, 43 spherical reflective markers with a diameter of 12.5 mm were affixed to the participant’s skin using double-sided adhesive tapes. A comprehensive description of the markers, including their labels and positions, can be found in Table 1, Figure 2 and Figure 3.

To ensure accurate data collection, the Vicon system underwent calibration for each participant at the start of the recording session. The participants maintained fixed, static T-positions at the beginning and end of each trial. The data from each trial were recorded at a frequency of 120 Hz. To mitigate noise and marker flickering during the trials, the recorded data were subjected to post-processing using Nexus 1.8.5 software by Vicon (Denver, CO, USA) [16]. This post-processing was performed on a computer running Windows 7, equipped with an Intel Core i5 processor, a 250 GB hard disk, and 32 GB of RAM.

The data collection process involved the utilization of a video camera, where videos were recorded at a frame rate of 25 frames per second. To ensure anonymity, each video underwent post-processing using video editor software. It is important to note that the videos are not included in this dataset. Their purpose was solely to ensure the accuracy of motion capture.

### 3.2. Participants

A total of 10 healthy adults participated in the data collection process, consisting of 4 men and 6 women. Among the participants, nine had dominant right hands, while one had a dominant left hand. Detailed demographic information about the participants is presented in the accompanying Table 2.

The average age of the participants was 28 years (SD = 8.56 years), with an average height of 164.32 cm (SD = 5.69 cm) and an average body mass of 66.10 kg (SD = 10.48 kg). Individuals with limited or no experience working with motion capture systems, including students and researchers, were recruited to ensure diverse participation in the study.

Ethical considerations were addressed by obtaining approval from the Institutional Review Board of the University of South Florida. Prior to data collection, all participants provided informed consent after receiving comprehensive information about the study and its procedures (IRB number: 004898).

### 3.3. Experimental Setup 

In this section, we will explore a dataset comprising nine carefully chosen daily living activities, along with the associated objects employed to perform these activities.

#### 3.3.1. Activities of Daily Living Tasks and Procedure

The dataset includes a total of nine activities of daily living (ADL) and eight range of motion (ROM) tasks [17]. As this data collection primarily centers on upper body movements, the selection of ADL tasks is based on prior work by the authors of references [18,19,20]. These activities focus on the movement of joints and how humans interact with objects while sitting or standing.

Participants were instructed to perform each task either in a standing position or while seated on a chair positioned behind a table with a height of approximately 88 cm. Prior to recording, the Vicon system underwent calibration for each participant to ensure accurate measurements. Two repetitions of each action were recorded, while participants maintained a fixed, static position at the start and end of each trial. 

Overall, a total of 18 demonstrations of ADL and 16 ROM were collected from each participant, resulting in a combined total of 340 demonstrations. The duration of each recording ranged between five to fifteen seconds.

#### 3.3.2. Objects

To capture human motion during the execution of activities of daily living (ADL) tasks, a set of seven natural household objects was employed. These objects were carefully selected to resemble the items commonly encountered in real-life scenarios closely. For comprehensive information regarding each object, including its weight and dimensions, please refer to Table 3. The dataset does not include information regarding object markers.

## 4. Data Post Processing

### 4.1. File Formats and Organization

**Figure 4:**
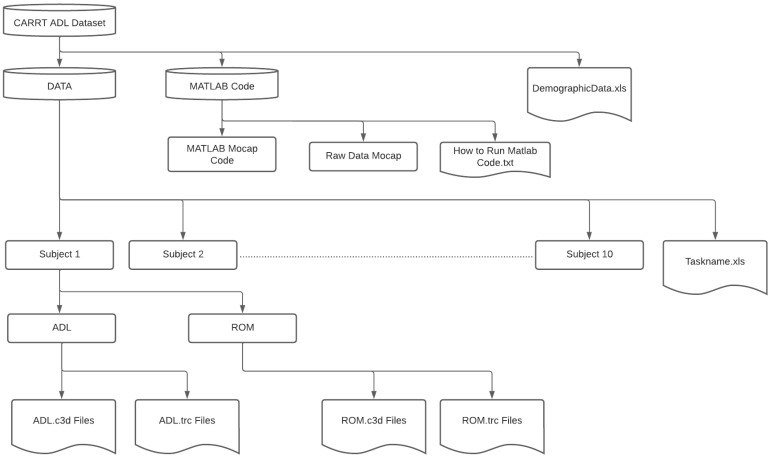
Dataset file schematic.

This section aims to present a comprehensive overview of the key software components involved in the study, including OpenSim, the MATLAB Robotic Human Upper Body Model (RHUBM), and the dataset organization. The dataset comprises 426 .c3d motion files and 426 .trc files specifically tailored for OpenSim. The Demographic Data .xls file contains essential participant information, such as ID, age, gender, dominant hand, weight, and body dimensions, which can be useful to create the scaled model in OpenSim. Additionally, the dataset includes seven MATLAB files, which comprise the RHUBM model of each subject, and 215 .xls joint angle files specifically tailored for MATLAB. Instructions on running the MATLAB code can be found in the accompanying text file. Please refer to Table 4 and Figure 4 for more detailed insights into the data content and format.

### 4.2. OpenSim Musculoskeletal Model

The musculoskeletal models offer a non-invasive approach to investigating human movement. In this dataset, we employed the full-body musculoskeletal model developed by Rajagopal et al. [21] to generate subject-specific joint angles for each task. By utilizing the OpenSim scaled model option, we created a customized model that accurately represents the individual’s anatomy. The skeletal structure of the model consisted of 22 articulating rigid bodies, including the pelvis, femurs, patella’s, tibia/fibulas, talus, calcaneus, and toes, to depict the lower body. Similarly, the upper body was represented by the combined head and torso and the humerus, ulna, radius, and hand for both sides. In total, the model encompassed 20 degrees of freedom in the lower body, accounting for the pelvis and each leg, and 17 degrees of freedom in the torso and upper body, incorporating the lumbar joint and each arm. The dataset offers comprehensive joint angles for the entire body. However, our primary research focus revolves around upper body movements specifically during activities of daily living. The focus is placed on analyzing and documenting the joint coordinates specifically related to the upper body. For a detailed understanding of each joint coordinate system, please refer to the work by Rajagopal et al. [21].

The head and torso were represented as a single rigid segment connected to the pelvis, with the orientation of the torso relative to the pelvis described using torso fixed ZXY rotations (representing lumbar extension, lateral bending, and rotation, respectively). The connection of the humerus with the torso was facilitated by a ball-and-socket joint, and the orientation of the right humerus in relation to the torso was determined by humerus-fixed ZXY rotations (representing shoulder flexion, adduction, and rotation, respectively). The ulna is linked to the humerus via a pin joint at the elbow, while forearm pronation was modeled by a pin joint connecting the radius and ulna. The hand was connected to the radius through a two-degree-of-freedom universal joint, with the orientation of the hand with respect to the radius described by hand-fixed ZX rotations (representing wrist flexion and ulnar deviation, respectively). It should be noted that this model focused primarily on capturing the overall motion of the torso and upper extremities using the OpenSim inbuild inverse kinematics toolbox and did not account for the complex kinematics of scapular motion or spinal bending.

### 4.3. MATLAB Robotic Toolbox Model

**Figure 5:**
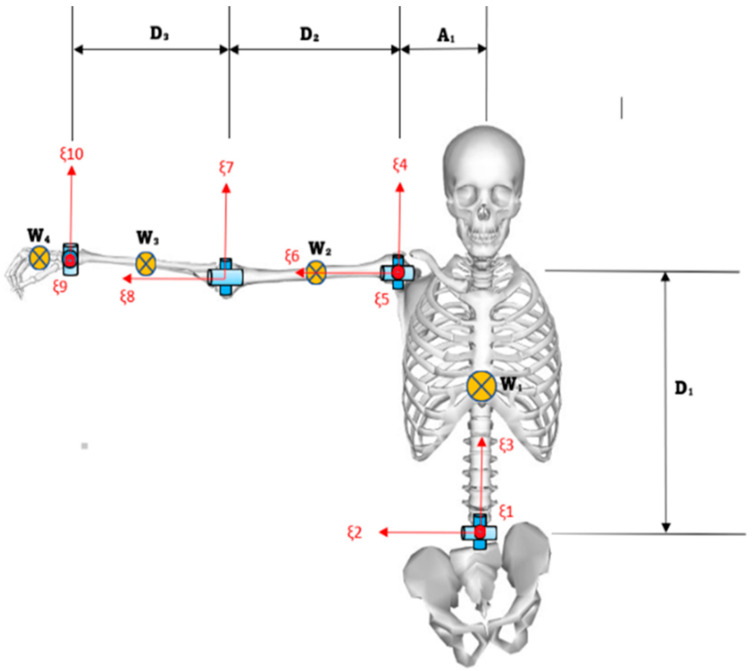
RHUBM Model.

In this dataset, the authors have provided only the upper body model designed explicitly for the Matlab environment based on the OpenSim model discussed in the above section. The upper body robot model was constructed using a rigid kinematic chain based on Denavit-Hartenberg (D-H) parameters [22]. The upper body model consists of 17 Degrees of Freedom (DoFs) for each subject. These DoFs include three for the torso (representing lumbar lateral bending, extension and rotation, respectively) [23], three for each shoulder joint (representing shoulder flexion, adduction, and rotation, respectively), two for each elbow joint (represent elbow flexion and forearm pronation), and two for each wrist joint (representing wrist flexion and ulnar deviation, respectively). The complete set of parameters used to create the links of the RHUBM is presented in Table 5, Table 6 and Table 7 and Figure 5. 

In the RHUBM, the vertical length of the torso is denoted as D1, and the horizontal length of the torso is represented as A1. The upper arm length is measured from the shoulder center to the elbow center, while the forearm length is measured from the elbow center to the wrist center. A2 denotes the upper arm length from shoulder to elbow center, and D3 represents forearm length. Additionally, the length of the hand, denoted as D4, is measured from the center of the wrist to the center of the palm. The mass of each body segment is expressed as a percentage of the total body mass [24]. Graphic descriptions of these parameters used in the RHUBM are provided in Figure 5.

## 5. Conclusions

In this study, we present a dataset consisting of human demonstrations capturing the activities of daily living and range of motion tasks. The dataset was recorded using the Vicon motion capture system and is specifically designed to provide comprehensive information for learning and analyzing human daily living activities for researchers focusing on studying various performance metrics that humans subconsciously optimize during task execution. It comprises nine different ADL actions and eight range of motion actions performed by ten subjects, with up to two repetitions of each action variation. This yields a total of 340 human demonstrations.

The dataset aims to facilitate the learning and generalization of object manipulation actions, which are crucial for analyzing various performance metrics optimized by humans during task performance. Using Vicon motion capture data, a subject-specific full-body model was constructed within the OpenSim framework, enabling the computation of joint angles. The Robotic toolbox developed by Peter Corke [25] played a crucial role in building a subject-specific Matlab-based upper body model. Additionally, a MATLAB-based library was created, leveraging OpenSim joint angles as a foundation, with the potential to support future research work. These tools and resources are intended to support research in learning performance criteria from human observation and can contribute to various research directions, such as motion primitive learning and trajectory planning.

In our future work, we plan to expand the dataset by including more actions and their variations from additional subjects. We also aim to incorporate bimanual tasks and a full-body MATLAB-based kinematic model to analyze the dynamics of the entire body. This will enable better trajectory planning for humanoid robots.

## Figures and Tables

**Table 1:** Marker description.

Name	Marker Placement
T1	Spinous Process; 1st Thoracic Vertebrae
T10	Spinous Process; 10th Thoracic Vertebrae
CLAV	Jugular Notch
STRN	Xiphoid Process
LBAK	Middle Of Left Scapula (Asymmetrical)
R/LASI	Right/Left Anterior Superior Iliac Spine
R/LPSI	Right/Left Posterior Superior Iliac Spine
R/LGT	Right/Left Greater Trochanters
R/LSHOA	Anterior Portion of Right/Left Acromion
R/LSHOP	Posterior Portion of Right/Left Acromion
R/LUPA	Right/Left Lateral Upper Arm
R/LELB	Right/Left Lateral Epicondyle
R/LELBM	Right/Left Medial Epicondyle
R/LFRA	Right/Left Lateral Forearm
R/LWRA	Right/Left Wrist Radial Styloid
R/LWRB	Right/Left Wrist Ulnar Styloid
R/LFIN	Dorsum Of Right Hand Just Proximal To 3rd Metacarpal Head
R/LTHI	Right/Left Thigh
R/LLFC	Right/Left Lateral Epicondyle of Femur
R/LMFC	Right/Left Medial Epicondyle of Femur
R/LTBI	Right/Left Tibia Interior
R/LLMAL	Right/Left Lateral Malleolus
R/LCAL	Right/Left Calcaneus
R/LTOE	Right/Left Toe

**Table 2:** Subject demographic information.

Subject	Gender	Dominant Hand	Height (cm)	Body Weight (Kg)
Subject 1	Male	R	162.5	60
Subject 2	Male	R	165.09	65
Subject 3	Female	R	160.02	58
Subject 4	Male	R	172.72	86
Subject 5	Female	R	152.4	58
Subject 6	Female	R	162.5	68
Subject 7	Female	R	165.09	51
Subject 8	Female	R	162.5	79
Subject 9	Male	L	172.72	60
Subject 10	Female	R	167.64	76

**Table 3:** Activities of daily living tasks. (* NA = Not Applicable).

ADL Task Name	Abbreviation	Object Used	Object Weight (Kg)	Object Size (m)
Brushing Hair	BH	Hairbrush	0.02	0.243 × 0.081 × 0.0381
Drinking From a Cup	FPC	Plastic Cup	0.02	0.053 × 0.053 × 0.109
Opening a Lower-Level Cabinet	OCL	* NA	NA	NA
Opening a Higher-Level Cabinet	OCH	NA	NA	NA
Picking Up the Box	PB	Carboard Box	0.52	0.457 × 0.356 × 0.305
Picking Up the Duster and Cleaning	PDC	Cleaning Duster	0.18	0.356 × 0.051 × 0.076
Picking Up an Empty Water Jug	PEWJ	1 Gallon Water Jug	0.90	0.15 × 0.15 × 0.269
Picking Up a Full Water Jug	PFWJ	1 Gallon Water Jug	3.79	0.15 × 0.15 × 0.269
Picking Up a Water Jug and Pouring	PWJ	1/2 Gallon Water Jug and Plastic Cup	2.55	0.191 × 0.105 × 0.289

**Table 4:** File formats.

Dataset	Format	Number of Files
Participant Data	.trc	426
Vicon Data	.c3d	426
Matlab Raw Data	.xls	215
Matlab Code	.M	7
Demographic Data and Task Name	.xls	2

**Table 5:** D-H parameters of RHUBM model.

i	αi-1 (deg)	ai-1 (m)	di (m)	Ɵi (deg)	Joint
1	0	0	0	90 + Ɵ1	Torso Lateral Flexion
2	90	0	0	90 + Ɵ2	Torso Flexion/Extension
3	−90	0	0	−90 + Ɵ3	Torso Rotation
4	0	A1	D1	Ɵ4	Shoulder Flexion/Extension
5	−90	0	0	−90 + Ɵ5	Shoulder Abduction/Adduction
6	−90	0	D2	Ɵ6	Shoulder Rotation
7	−90	0	0	180 + Ɵ7	Elbow Flexion
8	−90	0	D3	90 + Ɵ8	Elbow Pronation/Supination
9	−90	0	0	90 + Ɵ9	Wrist Flexion/Extension
10	−90	0	0	180 + Ɵ10	Wrist Abduction/Adduction

**Table 6:** Segment weight.

	Weight (Kg)	Segment
W1	0.551 × Body Weight	Torso
W2	0.0325 × Body Weight	Upper Arm
W3	0.0187 × Body Weight	Lower Arm
W4	0.0065 × Body Weight	Hand

**Table 7:** Subject segment dimensions.

Subject	W1 (Kg)	D1 (m)	W2 (Kg)	A1 (m)	W3 (Kg)	D2 (m)	W4 (Kg)	D3 (m)
Subject 1	29.820	0.435	1.680	0.200	0.960	0.270	0.360	0.265
Subject 2	32.305	0.432	1.820	0.280	1.040	0.280	0.390	0.254
Subject 3	28.826	0.368	1.624	0.140	0.928	0.300	0.348	0.250
Subject 4	42.742	0.457	2.408	0.200	1.376	0.318	0.516	0.265
Subject 5	28.826	0.356	1.624	0.190	0.928	0.254	0.348	0.228
Subject 6	33.796	0.356	1.904	0.203	1.088	0.267	0.408	0.254
Subject 7	25.347	0.432	1.428	0.200	0.816	0.254	0.306	0.229
Subject 8	39.263	0.457	2.212	0.200	1.264	0.254	0.474	0.228
Subject 9	29.820	0.435	1.680	0.200	0.960	0.270	0.360	0.265
Subject 10	37.772	0.432	2.128	0.200	1.216	0.254	0.456	0.228

## Data Availability

The data utilized in this review article are available in the sources cited and can be accessed through web searches or by contacting the authors of the original studies.
